# Association of dietary inflammatory index and refractive status in American adolescents: NHANES 1999–2008

**DOI:** 10.3389/fmed.2024.1511662

**Published:** 2024-12-05

**Authors:** Xiaodong Chen, Xuewei Li, Ningda Xu, Jiarui Li, Qianwen Guo, Heng Miao, Kai Wang, Lvzhen Huang

**Affiliations:** ^1^Department of Ophthalmology, Peking University People's Hospital, Eye Diseases and Optometry Institute, Beijing, China; ^2^Beijing Key Laboratory of Diagnosis and Therapy of Retinal and Choroid Diseases, Beijing, China; ^3^College of Optometry, Peking University Health Science Center, Beijing, China; ^4^Institute of Medical Technology, Peking University Health Science Center, Beijing, China; ^5^Shenzhen Eye Hospital, Shenzhen, Guangdong, China

**Keywords:** dietary inflammatory potential, myopia, adolescents, spherical equivalent, NHANES

## Abstract

**Background:**

Several nutrients have been found to be associated with the prevalence of myopia, and the role of dietary patterns in influencing myopia risk has recently garnered significant attention. We aim to explore the relationship between the Dietary Inflammatory Index (DII) and refractive status in adolescents.

**Methods:**

Data from 7,331 participants were analyzed from the 2005–2008 US National Health and Nutrition Examination Survey (NHANES). Smooth curve fitting and linear regression analysis were used to explore both non-linear and linear relationships between DII and spherical equivalent refraction (SER). The threshold effect of DII on SER was analyzed using a two-piecewise linear regression model.

**Results:**

DII was connected with a lower SER, indicating a shift toward myopia (*β*: –0.0586; 95% CI: −0.1109 to −0.0063; *p* < 0.05). Compared to the first quartile, the third quartile (*β*: –0.2512; 95% CI: −0.4952 to −0.0072; *p* < 0.05) and the fourth quartile (*β*: –0.2905; 95% CI: −0.5030 to −0.0780; *p* < 0.01) were significantly associated with a lower SER. Smooth curve fitting revealed a non-linear relationship between DII and SER, with a turning point at 0.81. For DII values below 0.81, there was no significant association with SER (*β*: –0.0450; 95% CI: −0.0272 to −0.1173; *p* > 0.05). However, for DII values ≥0.81, a significant association with lower SER was observed (*β*: –0.1197; 95% CI: −1.1722 to −0.0672; *p* < 0.01).

**Conclusion:**

These findings indicate that a higher DII (≥0.81) may contribute to the progression of myopia. This study highlights the potential for dietary recommendations in myopia prevention. Prospective studies are required to validate these findings and establish causal relationships.

## 1 Introduction

Myopia is a common eye condition that usually develops during childhood or early adolescence and is considered to be caused by genetic and environmental factors (1, 2). The prevalence of myopia is rising significantly worldwide. A meta-analysis of 145 studies predicted that by 2050, approximately 50% of the global population may be affected by myopia, with 10% of them suffering from high myopia (3). High myopia substantially increases the risk of ocular complications such as macular degeneration and glaucoma (4–6), leading to a considerable global socio-economic burden (2). Identifying risk factors is, therefore, crucial for preventing the development of myopia.

Diet is believed to play a role in the development of myopia (7, 8). Some studies have explored the association between many specific nutrients and myopia risk (9–12), but the results remain inconsistent. Some researchers have proposed that Western dietary patterns may be involved in the development of myopia (13). Nevertheless, few studies explored the connection between specific dietary patterns and myopia risk. Interestingly, the prevalence of myopia is reported to be very low among undisturbed hunter-gatherer populations, possibly due to their phytochemical-rich diet (14). Additionally, Yin et al. identified two dietary patterns—derived using principal component analysis with orthogonal rotation—that could reduce the risk of myopia. These patterns were characterized by high intakes of fruits, eggs, vegetables, dairy, and other nutrient-rich foods (15).

Although the exact mechanism underlying myopia pathogenesis remains unclear, inflammation has been identified as a key contributing factor (16, 17). The Dietary Inflammatory Index (DII) is a grading system developed from approximately 2,000 published articles across 11 countries (18). It assesses the inflammatory potential of 45 dietary parameters based on their effects on serum inflammatory biomarkers (19), with higher scores indicating greater inflammatory potential. High DII scores have been positively associated with various disorders in children, such as insulin resistance and asthma (20, 21).

To date, no study has explored the correlation between dietary inflammatory potential and refractive status. Therefore, this study aimed to assess this relationship in American adolescents aged 12 to 19 based on data collected from the United States National Health and Nutrition Examination Survey (NHANES).

## 2 Methods

### 2.1 Study population

The data for this cross-sectional study were obtained from NHANES, a series of interviews and examinations designed to represent the US population. All participants provided informed consent, and the protocols were approved by the National Center for Health Statistics board, eliminating the need for additional ethics approval.

**Figure 1:**
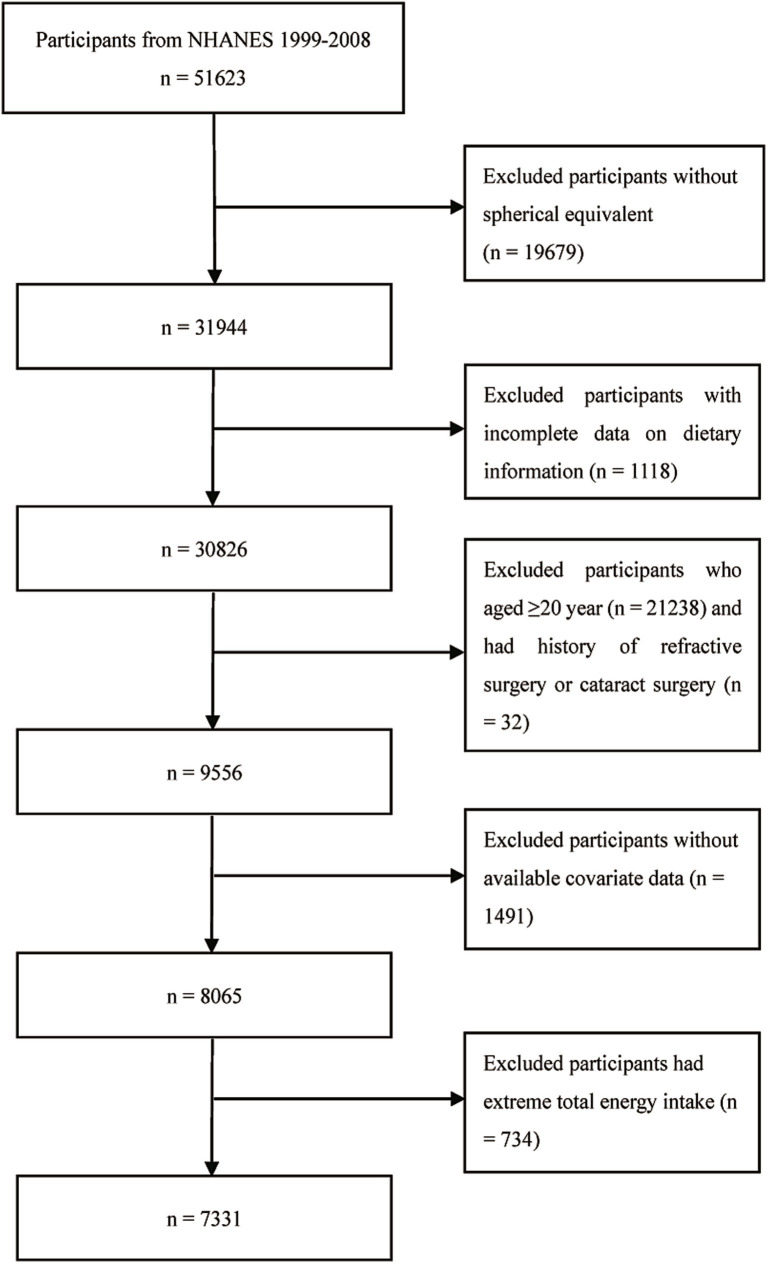
Selection of study population.

A total of 51,623 participants were initially enrolled in the 1999–2008 NHANES waves. Participants with incomplete spherical equivalent (SER) data (*n* = 19,679) and missing DII data (*n* = 1,118) were excluded. Additionally, those aged ≥20 years (*n* = 21,238), those with a history of refractive or cataract surgery (*n* = 32), and those with unavailable covariate data (*n* = 1,491) were removed from the analysis. Participants with extreme energy intake values (<800 or > 4,200 kcal/day for men and < 600 or > 3,500 kcal/day for women), as recommended by Willett in *Nutritional Epidemiology* (22), were also excluded (*n* = 734). Ultimately, 7,331 participants aged 12–19 years were included in the final analyses (Figure 1).

### 2.2 Assessment of refractive status

The sphere, cylinder, and axis length (average from three median measurements) of both eyes were objectively examined using non-cycloplegic refraction with the Nidek Auto Refractor Model ARK-760. The spherical equivalent refraction (SER) was calculated as the sphere plus half of the cylinder. Due to the strong correlation between SER values of the right and left eyes (Spearman correlation coefficient = 0.90), only the right eye was used for the analysis.

### 2.3 Dietary inflammatory index

The DII is a composite scoring system developed by Shivappa et al. to evaluate the inflammatory potential of dietary nutrient consumption on inflammatory biomarkers (18). The DII calculation in this study used 27 nutrients available from 24-h dietary recalls in NHANES 1999–2008, including riboflavin, alcohol, *β*-carotene, polyunsaturated fatty acids, caffeine, carbohydrates, cholesterol, omega-3 fatty acids, total energy, thiamin, fiber, folic acid, iron, magnesium, zinc, selenium, monounsaturated fatty acids, niacin, total fat, omega-6 fatty acids, protein, saturated fat, and vitamins A, B6, B12, C, and E. Pro-inflammatory nutrients were assigned to positive inflammatory effect scores, while anti-inflammatory nutrients received negative scores.

The total DII score was calculated as the sum of the scores for these 27 nutrients. Previous studies confirmed that predictive ability was maintained when using 27 or 28 of the 45 total DII parameters (19, 23). For this analysis, the DII was calculated using dietary intake data from the first 24-h dietary recall.

### 2.4 Covariates

Covariates included in the analysis to control for potential confounding variables were age, family income-poverty ratio, gender, race, education level, and total energy intake. The body mass index (BMI) z-scores were calculated based on the CDC growth charts (24). According to the previous studies, tobacco exposure was defined using serum cotinine levels as follows: (a) <0.05 ng/mL, unexposed; (b) 0.05–3 ng/mL, passive exposed; and (c) ≥ 3 ng/mL, active exposed (25, 26).

### 2.5 Statistical analyses

Analyses were conducted following the CDC guidelines for weighted oversampling data. Participant characteristics were displayed as means ± standard deviation (SD) or percentages. Linear regression analysis was used to determine *β* coefficients and 95% confidence intervals (CI). DII was analyzed both as a continuous variable and by quartiles.

The crude model included no covariate. Model 1 adjusted for age, sex, race, BMI z-score, education level, tobacco exposure, and family income-poverty ratio. Model 2 was further adjusted for total energy intake, based on model 1. Weighted generalized additive models and smooth curve fittings were used to explore the non-linear association between DII and SER.

The threshold effect of DII on SER was analyzed using a two-piecewise linear regression model. Sensitivity analyses were conducted to ensure robustness. First, missing covariate data were imputed using the “mice” R package, generating five imputed datasets, with one dataset used for further analysis (sensitivity analysis i) (27). Second, since serum vitamin D levels may be a potential confounding factor for refractive status (28) but were not measured in the 1999–2000 NHANES cycle, serum vitamin D levels were included in the model using data from the 2001–2008 NHANES cycles (sensitivity analysis ii). Third, extreme SE values (< −15 D) were excluded (sensitivity analysis iii). Subgroup analyses were stratified by age (12–15 or 16–19), sex (male or female), and race (Mexican American, other Hispanic, non-Hispanic White, non-Hispanic Black, or others). All analyses were conducted using R 4.3.2. Statistical significance was defined as a *p* value of <0.05.

## 3 Results

### 3.1 Participants

The mean age was 15.43 ± 2.26 years, and 49.5% were women. The mean SER of participants was −0.83 ± 1.88 D, ranging from −20.75 D to +9.5 D. Significant variations in age, sex, BMI z-score, family income-poverty ratio, education level, tobacco exposure, and total energy intake were notable. Participants in the third and fourth quartile groups had lower SER than those in the first and second quartile groups (Table 1).

**Table 1:** Characteristics of participants according to the quartile of DII.

Characteristics	Overall	DII				*p*-value
		Quartile 1	Quartile 2	Quartile 3	Quartile 4	
Number of participants	7,331	1,804	1,792	1,880	1,855	
Age, years	15.43 (2.26)	15.71 (2.28)	15.49 (2.27)	15.29 (2.23)	15.25 (2.24)	0.004
Sex						<0.001
Male	3,621 (50.5)	1,087 (61.8)	947 (54.1)	888 (46.8)	699 (39.4)	
Female	3,710 (49.5)	717 (38.2)	845 (45.9)	992 (53.2)	1,156 (60.6)	
Race						0.172
Mexican American	2,456 (11.5)	689 (13.2)	618 (11.8)	588 (11.0)	561 (9.9)	
Other Hispanic	382 (5.8)	108 (7.0)	92 (6.4)	91 (5.2)	91 (4.6)	
Non-Hispanic White	1,975 (62.8)	482 (62.3)	469 (62.2)	505 (63.0)	519 (63.5)	
Non-Hispanic Black	2,223 (13.9)	457 (12.0)	540 (13.4)	623 (15.5)	603 (14.8)	
Others	295 (6.1)	68 (5.5)	73 (6.2)	73 (5.3)	81 (7.2)	
BMI z-score	0.52 (1.13)	0.41 (1.15)	0.49 (1.15)	0.51 (1.12)	0.67 (1.09)	0.002
Family income-poverty ratio	2.54 (1.63)	2.70 (1.67)	2.49 (1.63)	2.53 (1.61)	2.42 (1.59)	0.019
Education level						0.011
Less than 9th Grade	4,339 (56.9)	978 (50.5)	1,037 (56.2)	1,164 (60.7)	(60.2)	
High school	2,004 (26.8)	532 (29.9)	506 (28.0)	484 (24.8)	(24.4)	
High school graduate and above	988 (16.4)	294 (19.7)	249 (15.8)	232 (14.6)	(15.4)	
Tobacco exposure						<0.001
Unexposed	2,894 (39.9)	815 (44.8)	748 (43.4)	725 (39.2)	(32.4)	
Passive exposed	3,280 (41.1)	712 (38.2)	767 (38.4)	843 (40.1)	(47.8)	
Active exposed	1,157 (18.9)	277 (17.0)	277 (18.2)	312 (20.7)	(19.9)	
SER, D	−0.83 (1.88)	−0.74 (1.80)	−0.72 (1.71)	−0.91 (1.91)	−0.94 (2.08)	0.062
Total energy intake, g/day	2117.79 (764.02)	2744.42 (685.07)	2260.90 (658.29)	1925.36 (585.87)	1540.24 (552.92)	<0.001

Data are presented as mean (SD) or percentage (%). BMI, body mass index; DII, dietary inflammatory index; SER, spherical equivalent refraction.

### 3.2 Association between DII and spherical equivalent refraction

**Figure 2:**
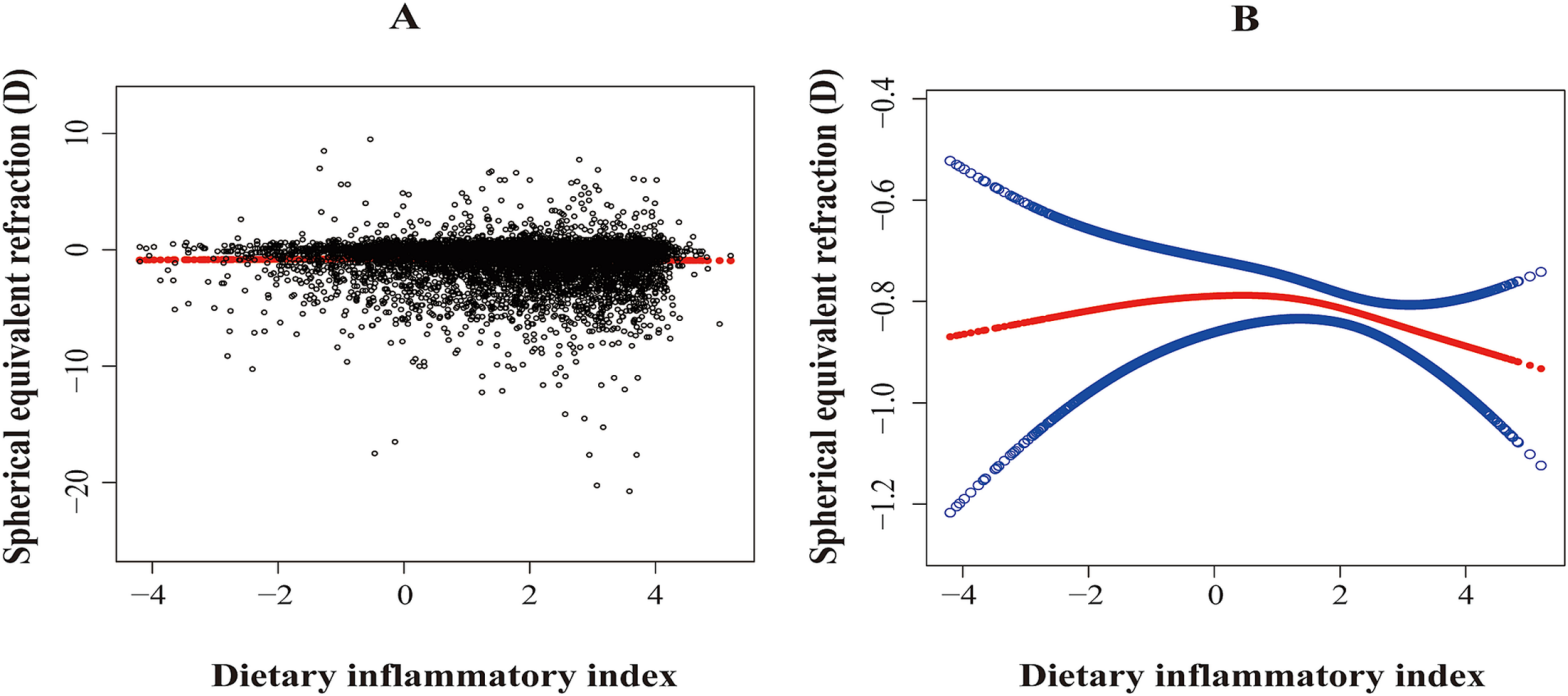
The association between dietary inflammatory index and spherical equivalent refraction (D). **(A)** Scatterplot: Each black dot represents a sample. **(B)** Red arcs indicate the smoothed curve fit between variables. The two blue bands represent the 95% confidence intervals of the fit values. The model was adjusted for age, sex, race, BMI z-score, family income-poverty ratio, education level, tobacco exposure, and total energy intake per day.

DII was associated with a lower SER, indicating a shift toward myopia (*β*: –0.0586; 95% CI: −0.1109 to −0.0063; *p* < 0.05). Compared to the first quartile, the third quartile (*β*: –0.2512; 95% CI: −0.4952 to −0.0072; *p* < 0.05) and the fourth quartile (*β*: –0.2905; 95% CI: −0.5030 to −0.0780; *p* < 0.01) were associated with a lower SER (Table 2). In addition, smoothed curve fitting revealed a non-linear relationship between DII and SER (Figures 2A,B).

**Table 2:** Logistic regression analysis of the association of dietary inflammatory index and spherical equivalent refraction (D).

	Crude, β (95% CI)	Model 1, β (95% CI)	Model 2, β (95% CI)
DII			
Continuous	−0.0419 (−0.0831, −0.0006)*	−0.0555 (−0.0991, −0.0119)*	−0.0586 (−0.1109, −0.0063)*
Quartile 1	Reference		
Quartile 2	0.0219 (−0.1487, 0.1891)	−0.0098 (−0.1759, 0.1563)	−0.0239 (−0.1897, 0.1420)
Quartile 3	−0.1738 (−0.3938, 0.0462)	−0.2273 (−0.4526, −0.0020)*	−0.2512 (−0.4952, −0.0072)*
Quartile 4	−0.1972 (−0.3652, −0.0292)*	−0.2556 (−0.4383, −0.0730)**	−0.2905 (−0.5030, −0.0780)**

CI, confidence interval. The crude model was adjusted for none. Model 1 was adjusted for age, sex, race, BMI z-score, family income-poverty ratio, education level, and tobacco exposure. Model 2 was adjusted for age, sex, race, BMI z-score, family income-poverty ratio, education level, tobacco exposure, and total energy intake per day. *p < 0.05, **p < 0.01.

After adjusting for all covariates, a two-piecewise linear regression model identified a turning point at 0.81. For DII < 0.81, there was no significant association between DII and SER (*β*: –0.0450; 95% CI: −0.0272 to −0.1173; *p* > 0.05). However, for DII ≥ 0.81, DII was significantly associated with a lower SER (*β*: –0.1197; 95% CI: −1.1722 to −0.0672; *p* < 0.01; Table 3).

**Table 3:** Threshold effect analysis of dietary inflammatory index on spherical equivalent refraction (D) using a two-piecewise linear regression model.

	β (95% CI)
Fitting by a two-piecewise linear model	
Inflection point	0.810
DII < 0.81	0.0450 (−0.0272, 0.1173)
DII ≥ 0.81	−0.1197 (−1.1722, −0.0672)**
Log-likelihood ratio	0.002

DII, dietary inflammatory index. The model was adjusted for age, sex, race, BMI z-score, family income-poverty ratio, education level, tobacco exposure, and total energy intake per day. *p < 0.05, **p < 0.01.

### 3.3 Subgroup analyses

After stratification by age, DII was associated with a lower SER in the older adolescents aged 16–19 subgroup (*β*: –0.1123; 95% CI: −0.1987 to −0.0270; *p* < 0.05). Stratification by sex revealed a significant association between DII and lower SER in the male subgroup (*β*: –0.0717; 95% CI: −0.1417 to −0.0001; *p* < 0.05). When stratified by race, DII was associated with a lower SER in the White subgroup (*β*: –0.1071; 95% CI: −0.1849 to −0.0294; *p* < 0.01). No significant interaction was observed (Table 4).

**Table 4:** Subgroup analysis of the association between dietary inflammatory index on spherical equivalent refraction (D).

	β (95% CI)	P for interaction
Age		0.072
12–15 (*n* = 3,797)	−0.0004 (−0.0636, 0.0710)	
16–19 (*n* = 3,534)	−0.1123 (−0.1987, −0.0270)*	
Sex		0.599
Male	−0.0717 (−0.1417, −0.0001)*	
Female	−0.0415 (−0.1076, 0.0244)	
Race		0.978
Mexican American (*n* = 2,456)	0.0058 (−0.0690, 0.0806)	
Other Hispanic (*n* = 382)	0.0724 (−0.0995, 0.2444)	
Non–Hispanic White (*n* = 1975)	−0.1071 (−0.1849, −0.0294)**	
Non–Hispanic Black (*n* = 2,223)	0.0235 (−0.0693, 0.1163)	
Others (*n* = 295)	0.0680 (−0.1371, 0.2731)	

DII, dietary inflammatory index. The model was adjusted for age, sex, race, BMI z-score, family income-poverty ratio, education level, tobacco exposure, and total energy intake per day. *p < 0.05, **p < 0.01.

### 3.4 Sensitivity analyses

Three sensitivity analyses showed comparable results with the main results (Table 5).

**Table 5:** Sensitivity analyses of the association between dietary inflammatory index on spherical equivalent refraction (D).

	Sensitivity i (*n* = 8,693)	Sensitivity ii (*n* = 5,679)	Sensitivity iii (*n* = 7,324)
DII			
Continuous	−0.0497 (−0.0990, −0.0005)*	−0.0628 (−0.1294, 0.0037)	−0.0516 (−0.0983, −0.0049)*
Q1			
Q2	−0.0263 (−0.1917, 0.1390)	−0.0869 (−0.2774, 0.1036)	−0.0443 (−0.2148, 0.1262)
Q3	−0.1765 (−0.3956, 0.0425)	−0.2537 (−0.5626, 0.0552)	−0.1857 (−0.3921, 0.0207)
Q4	−0.2330 (−0.4612, −0.0049)*	−0.2952 (−0.5600, −0.0304)*	−0.2484 (−0.4691, −0.0278)*

DII, dietary inflammatory index. Data was shown in β (95% CI). The model was adjusted for age, sex, race, BMI z-score, family income-poverty ratio, education level, tobacco exposure, and total energy intake per day in the sensitivity i. The model was adjusted for age, sex, race, BMI z-score, family income-poverty ratio, education level, tobacco exposure, total energy intake per day, and vitamin D in the sensitivity ii. *p < 0.05.

## 4 Discussion

This study is believed to be the first to assess the association between DII and myopia, indicating that a higher DII (indicating greater pro-inflammatory potential) was associated with a reduced SER, which reflected a shift toward myopia in American adolescents aged 12 to 19 years. Smooth curve fitting demonstrated an approximately inverted U-shaped relationship between DII and SER, with a turning point at 0.81. A two-piecewise linear model indicated that the negative association between DII and SER was significant only when DII > 0.81. Two sensitivity analyses proved the robustness of these results.

In 1958, Gardiner first proposed that healthy eating habits may prevent the progression of myopia (29). Studies have shown that a whole-grain intake of>50% is an independent protective factor against myopia (30), and a randomized cross-over trial demonstrated that a whole-grain-rich diet could reduce inflammation and body weight (31). Two dietary patterns characterized by high consumption of fruits, grains, vegetables, and potatoes or by high intake of aquatic products, meats, dairy, eggs, and legumes were associated with a lower myopia risk (15). These two patterns, resembling the Mediterranean diet, may possess anti-inflammatory properties (32).

Saturated fat, carbohydrate and cholesterol were the main pro-inflammatory contributors in the DII score system, and the three nutrients intake were reported to increase myopia risk (7, 9). It is reported that upregulated metabolisms of triglyceride and cholesterol may lead to axial length (AL) elongation and myopia (33, 34). Besides, an inverse relationship between AL and serum high-density lipoprotein cholesterol in Chinese children (35). Meanwhile, omega-3 and vitamin C intake were the main anti-inflammatory contributors in the DII score system, and the two nutrients’ intakes were reported to reduce myopia risk (9, 36). Two studies reported that the supplementation of omega-3 fatty acids could alleviate scleral hypoxia and suppress choroidal thinning, and thus slow the progression of myopia in mice (37, 38). This study indicated that a pro-inflammatory diet including high DII score (nutrients like saturated fat, carbohydrate and cholesterol) may promote myopia progression.

Inflammation is thought to increase myopia risk through mechanisms such as inducing scleral remodeling (16). Some studies have linked serum inflammatory biomarkers like C-reactive protein (CRP) and white blood cell (WBC) count to a higher prevalence of myopia (39, 40). Moreover, individuals with myopia have been found to exhibit elevated levels of inflammatory factors in the vitreous or aqueous humor, such as interleukin-6 and matrix metalloproteinase-2, indicating low-grade inflammation activation in the ocular microenvironment (41, 42).

In addition, intravitreal injections of anti-inflammatory drugs, such as ketorolac tromethamine and dexamethasone, have been shown to inhibit the development of myopia in chickens (43). Atropine has also been reported to downregulate inflammation in animal models of myopia (17). DII has been closely associated with serum inflammatory cytokines in adolescents (44). However, its relationship with inflammatory factors in aqueous humor or vitreous humor remains unclear. Further studies are warranted to investigate this connection and its potential implications for understanding myopia development.

This current study had several strengths, including a large sample size and strong national representativeness of the study population. However, some limitations in the analysis should be taken into rigorous consideration. First, the cross-sectional design of the study precludes the determination of causality. Second, although refractive error measurements were repeated three times to obtain the median value, they were assessed using non-cycloplegic refraction, which may introduce methodological errors. Third, dietary intake was based on a single 24-h recall interview, which could lead to recall bias. Fourth, while this study adjusted for numerous covariates, other potential confounding factors, such as genetic influences and time spent outdoors, were not accounted for.

## 5 Conclusion

In general, our results indicate that a higher DII (≥0.81), indicative of a pro-inflammatory diet, may increase the risk of myopia. This study highlights the importance of dietary recommendations for myopia prevention. However, further large-scale, well-designed studies are needed to confirm these findings.

## Data Availability

The original contributions presented in the study are included in the article/supplementary material, further inquiries can be directed to the corresponding authors.
